# Properties and Characterization of a PLA–Chitin–Starch Biodegradable Polymer Composite

**DOI:** 10.3390/polym11101656

**Published:** 2019-10-11

**Authors:** N. G. Olaiya, Indra Surya, P. K. Oke, Samsul Rizal, E. R. Sadiku, S. S. Ray, P. K. Farayibi, Md Sohrab Hossain, H. P. S. Abdul Khalil

**Affiliations:** 1 Department of Industrial and Production Engineering, Federal University of Technology, P.M.B.740 Akure, Nigeriapkfarayibi@futa.edu.ng (P.K.F.); 2 School of Industrial Technology, Universiti Sains Malaysia, 11800 Penang, Malaysia; 3 Department of Chemical Engineering, Universitas Sumatera Utara, Medan 20155, Indonesia; 4 Department of Mechanical Engineering, Universitas Syiah Kuala, Banda Aceh 23111, Indonesia; rizal@knt.mech.tut.ac.jp; 5 Department of Chemical, Metallurgical and Materials Engineering, Tshwane University of Technology, P.M.B. X680 Pretoria, South Africa; sadikur@tut.ac.za; 6 DST-/CSIR National Centre for Nanostructured Materials, Council for Scientific and Industrial Research, Pretoria 0001, South Africa; rsuprakas@csir.co.za

**Keywords:** biopolymer, starch, biodegradable, chitin, degradation

## Abstract

This paper presents a comparison on the effects of blending chitin and/or starch with poly(lactic acid) (PLA). Three sets of composites (PLA–chitin, PLA–starch and PLA–chitin–starch) with 92%, 94%, 96% and 98% PLA by weight were prepared. The percentage weight (wt.%) amount of the chitin and starch incorporated ranges from 2% to 8%. The mechanical, dynamic mechanical, thermal and microstructural properties were analyzed. The results from the tensile strength, yield strength, Young’s modulus, and impact showed that the PLA–chitin–starch blend has the best mechanical properties compared to PLA–chitin and PLA–starch blends. The dynamic mechanical analysis result shows a better damping property for PLA–chitin than PLA–chitin–starch and PLA–starch. On the other hand, the thermal property analysis from thermogravimetry analysis (TGA) shows no significant improvement in a specific order, but the glass transition temperature of the composite increased compared to that of neat PLA. However, the degradation process was found to start with PLA–chitin for all composites, which suggests an improvement in PLA degradation. Significantly, the morphological analysis revealed a uniform mix with an obvious blend network in the three composites. Interestingly, the network was more significant in the PLA–chitin–starch blend, which may be responsible for its significantly enhanced mechanical properties compared with PLA–chitin and PLA–starch samples.

## 1. Introduction

Polymers can be categorized into synthetic and non-synthetic, or biodegradable and non-biodegradable [1]. The series of studies on synthetic polymers have brought about their various excellent applications in manufacturing industries. However, there is still an increasing quest for new materials for the manufacturing of products. As expected, this is accompanied by peculiar challenges. The major problem with synthetic polymers is their disposal after use. The problem of disposal has become a significant issue as most synthetics are not degradable or only minimally degradable even when exposed to heat. Poly(lactic acid) (PLA) is a polyester and biodegradable polymer which is one of the most promising natural polymers that can be used for plastic packaging with similar properties as synthetic ones [2,3,4]. It is one of the most consumed natural polymers in industrial applications. It is sourced from starch by a direct condensation process. PLA degrades rapidly in suitable environments and can be recycled [5,6]. PLA has been used for several industrial manufacturing products, among which are packaging [7,8] and biomedical implants. The use of PLA as a packaging material or for biomedical implants is due to its limited mechanical strength. However, previous work on degradable polymers has revealed that PLA has great potential to replace some of the conventional synthetic polymers with improvement in its mechanical properties. In other to achieve this, the recent trend of research has focused on polymer blending and reinforcement. However, care is often taken to ensure that blending/reinforcement for improved mechanical properties does not tamper with the biodegradable nature of PLA.

Starch is a natural biopolymer which is abundantly available at low cost [9,10]. A blend of starch with PLA has been reported to have improved biodegradation but with reduced mechanical properties [11,12]. Specifically, the mechanical properties were reported to reduce with increasing starch content. The reduced mechanical properties are mainly due to weak adhesive forces between the molecules of PLA and starch. This poor adhesion has been attributed to the hydrophilic nature of starch compared with PLA, which is hydrophobic [4].

Chitin is abundantly available in shrimps, crabs [13,14], and crustaceans [15] and is rated as the second most abundant polymer. It is non-toxic and biodegradable which makes it useful in biomedical applications. In a particular study, it was reported that incorporating a chitin nanofiber with PLA resulted in good dispersion but minimal improvement in mechanical properties [16]. Therefore, further study was recommended with a low percentage of PLA in the chitin nanofiber blend to study the functional properties [16].

Literature established that for the blending of PLA, the percentage of the polymer blend should be a ratio 1:9 by weight of the blend to ensure good miscibility [17]. Several researchers have worked on either a PLA blend with starch or a PLA blend with chitin. Previous work on PLA–starch showed that starch can be used to improve the biodegradability of PLA, but there is a challenge of miscibility [18,19]. However, it has been reported [4] that the PLA–starch miscibility is enhanced by the use of multifunctional alcohol as a plasticizer, but with adverse effect on the tensile strength of the composite. Also, [1] stated in their work that the hygroscopic nature and impact strength of starch decreases with PLA increase, but the storage, stress–strain, and flexural moduli properties are enhanced. The work of [17] on the extrusion of PLA–starch showed that the interdependence of starch and PLA increased with a decrease in starch percentage, especially when the ratio of starch to PLA is less than 1:10 and the water absorption was reduced. The reinforcing effect of starch is established in [20], where work on PLA–starch using glycerol as a plasticizer and a compression molding method were performed. The stiffness and strength of the composite were enhanced with the use of a plasticizer. Chitin, on the other hand, is mainly used because it is cheap and bioabsorbable. It has several applications in biomedical engineering and has been found to have a positive effect on the mechanical properties of PLA.

Obviously, starch and chitin have been differently used by several researchers to enhance the mechanical and biodegradability of PLA [21,22,23,24], but their combined effect has not been studied. Also, the comparative effect of the blending of PLA–chitin, PLA–starch and PLA–chitin–starch on their mechanical properties has not been reported. Therefore, this study seeks to establish the combined effect of a starch–chitin blend on PLA, as well as to determine which of the blends (PLA–starch or PLA–chitin) gives a better result in terms of mechanical and degradation properties.

## 2. Materials and Methods 

### 2.1. Materials 

Commercial grade poly(lactic acid) (4032D) was supplied from Nature Works (Minnetonka, MN, USA) in pellet form. PLA (4032D) has excellent grease and oil resistance as well as optical, machinability, twist and dead fold. PLA has a melting point between 150–170 °C and a glass transition temperature of 60–65 °C. The tensile strength, yield strength, elastic modulus and impact strength of the PLA used are 53.5 MPa, 60 Mpa, 3500 Mpa and 2.99 kJ/m^2^, respectively. The average molecular weight of PLA (4032D) is 100,000 g/mole. It has a specific gravity of 1.24, melt flow rate (MFR), g/10 min (210 °C, 2.16 kg) of 7 and a melt density of 1.08 g/cc at 230 °C. Commercially available chitin was used (lifeline nutrients, Chicago, IL, USA) with a 90% degree of deacetylation. On the other hand, commercial high amylose starch from corn was supplied by Sigma Aldrich (Sigma Aldrich, Modderfontein, South Africa). The particle size distribution is presented in Table 1.

### 2.2. Composite Preparation

Poly(lactic acid) from Nature Works was ground into a powder using an AA 150 Power granulator (Pulian, Taichung, Taiwan). The ground PLA was then dried for 4 h at 60 °C in a Thermo Electron Luxor 50 Motan Desiccant Dryer (Motan, Überlingen, Germany) with dry air at 50 m^3^/h. The PLA powder was mixed with chitin and starch in the proportions shown in Table 2 using the HAAKE Rheomix OS Lab Mixers system. The mixtures were then extruded using a Thermo Electron Process 11 extruder (Thermo Fisher Scientific, Waltham, MA, USA) at a temperature range of 120 °C to 190 °C, extrusion speed of 201 rpm, 6.1 Nm torque and 35 bar pressure. The extruded filament was pelletized using a Thermo Scientific Varicut Pelletizer 11M (Thermo Fisher Scientific, Waltham, MA, USA). The composite pellets were moulded into test samples using the Carver Press (model 3851-0) (Carver, Wabash, IN, USA) at a temperature of 170 °C and a pressure of 2 pascals. The test samples were characterized for tensile strength, impact strength, and dynamic mechanical analysis (DMA). The morphology was observed using scanning electron microscopy (SEM). 

The composition variation for the PLA–starch composite was done based on previous studies [17,22]. It was stated that the tensile strength of PLA increased maximally when the percentage of starch in the blend was 10%. However, a further investigation of the mechanical properties of PLA–starch at less than 10% of starch blending was not reported. On the other hand, a study on a PLA–chitin composite showed that with a lower percentage of chitin (1% and 5% ), the mechanical strength was improved compared to when the percentage of chitin was higher than 10% [25]. A similar result was also reported by [16] for a PLA–chitin nanofiber with 2% and 5% chitin.

The composition variation for the percentage proportion by weight of PLA–chitin–starch in this study is shown in Table 2. The aim is to study the effect of composition variation on mechanical strength. Three types of composite blends with four sets of samples were produced to compare the binary and ternary blending of chitin and/or starch with PLA as shown in Table 2.

### 2.3. Characterization 

#### 2.3.1. Tensile Test

Tensile samples were prepared according to the American Society for Testing and Materials ASTM D3039 method for plastic using the Carver Press machine. The tensile test method was used with standard dimensions and loaded using an Instron Universal Testing Machine (model 5966) (Instron, Norwood, CO, USA) with a force capacity of 10 kN for five replicates per sample. 

#### 2.3.2. Impact Test

The impact test was done using Ceast Resil Impactor 7181 (Corporate Consulting, Service and Instruments (CCSi), Akron, OH, USA), which uses an instrumented hammer. The test is to determine the energy absorbed in breaking. The energy required to break the specimen is obtained from the loss of energy in the pendulum. This energy is simply the difference in potential energy of the hammer before and after the impact. The values are reported in terms of absorbed energy per unit cross-sectional area at the notch (J/m^2^). The dimensions of an ASTM D256 standard specimen was used. 

#### 2.3.3. Dynamic Mechanical Analysis

Dynamic mechanical analysis was used in this study for structural and mechanical analysis of polymers, and to compare the miscibility of the polymeric blend system. Dynamic mechanical analysis shows the mechanical properties of the composites at different temperatures. This was done using a PerkinElmer Dynamic Mechanical Analyzer (DMA 8000) (PerkinElmer Inc., OH, USA). Sample shape and dimension were prepared according to the ASTM D4065 standard for polymer composites at a frequency of 10 Hz and a temperature range of −50 °C to 150 °C.

#### 2.3.4. Thermogravimetry Analysis Test

The thermal properties of the samples were measured using a PerkinElmer TG-IR-GCMS Interface Q500, TA Instruments (PerkinElmer Inc., OH, USA). The result was plotted and analyzed with TA universal analysis software (TA instruments, Lukens Drive New Castle, PA, USA). Sample quantities between 20 mg and 21 mg were heated at a rate of 10 °C/min from room temperature to 600 °C under air.

#### 2.3.5. Microstructural Analysis

The microstructure of the fractured surface (after the tensile test) of samples were thoroughly investigated using SEM. The samples were carbon-coated, and microstructural examination was performed on carbon-coated samples using a JEOL Field Emission Scanning Electron Microscope (JSM7500F, JEOL, Boston, MA, USA).

## 3. Results and Discussion

### 3.1. Tensile Properties of the Composite

Table 3 shows the values of the tensile modulus, yield strength and tensile strength for each of the composites (PLA–chitin–starch, PLA–chitin and PLA–starch). The pure PLA used in this research has a tensile strength in of 53.5 MPa. Generally, it can be seen that for each of the sets, the blend with both chitin and starch has the highest tensile strength, followed by that of chitin. The polymer composite sample with 94% PLA–3% chitin–3% starch has the best tensile strength of all the samples. Also, it can be deduced that the tensile strength reduces with an increase in PLA percentage, similar to what was previously reported [4,17]. The work of previous researchers has established that the tensile strength of the PLA–starch composite reduces with increasing starch content above 10%. However, there is no literature to account for the optimum percentage composition of starch below 10% in a PLA–starch blend. The tensile graph established that PLA–starch has the highest tensile strength at 96% PLA–4% starch, which is probably due to a more even distribution of the starch in the PLA. This cannot be fully established as the variation from the deviation is quite significant compared to the other two sets of values Also, for PLA–chitin, the tensile strength is found to decrease with a reduction in chitin content, which correlates with previous studies [16,25]. This shows that chitin has good dispersion in PLA.

The yield strength of the composite samples of the blend of the three polymers (PLA–chitin–starch) has the highest value compared with PLA–starch and PLA–chitin. PLA–starch and PLA–chitin show the same trend as that of the tensile result. This result of yield strength for PLA–chitin is in accordance with [25]. However, the trend of yield strength for PLA–starch is a newly established trend when the percentage of starch is less than 10%.

The value of the elongation shows that the value for PLA–chitin–starch falls between that of PLA–chitin and PLA–starch as shown in Table 3. The value of the elongation reduces with the addition of starch. This can be explained as the addition of starch increases the brittleness of PLA, while chitin reduced it. This aligns with reports in the literature [26,27,28].

The Young’s modulus value is a critical factor for high strength applications. The value of the Young’s modulus follows a different trend from the tensile strength and elongation. There is no significant trend with variation in composition for PLA–starch–chitin and PLA–starch, but samples have the highest values at 96% PLA–2% chitin–2% starch and 96% PLA–4% starch. However, it is worthy of note that the value of Young’s modulus for the PLA–chitin composite increases with decreasing chitin content. The results shown in [16] and [25] have a similar trend for the PLA–chitin composite for a percentage of chitin between 1% to 10%.

### 3.2. Impact Test

**Figure 1:**
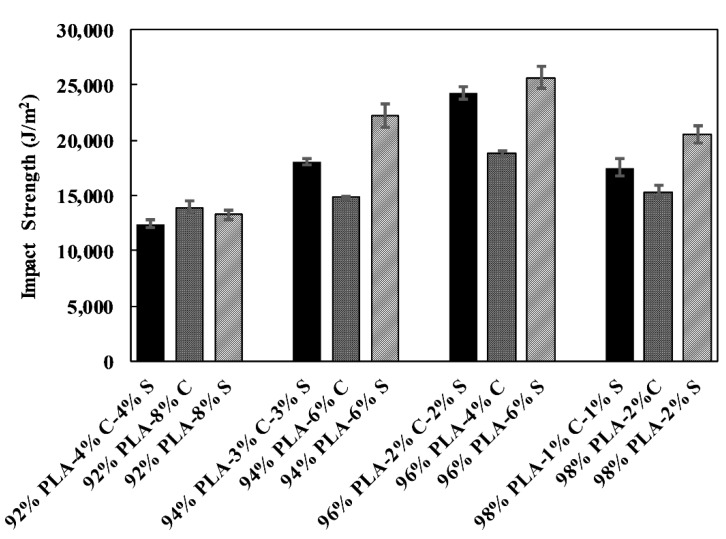
Comparative impact strength.

The impact strength is a measure of the resistance of a material to the sudden force. The result of the impact strength for each of the samples is shown in Figure 1. The impact strength shows no specific pattern except that the impact strength increases with an increase in PLA percentage with a sudden decline at 98%. The drop probably means that the blending process causes distortion rather than a toughening effect. Notably, the graph shows 96% sets as having the highest impact strength, and this shows an adequate toughening percentage of the blend. The effect of starch on the impact strength of the samples is more significant in each of the sets of samples. This is as a result of the brittle nature of the PLA–starch composite as reported previously [17,27]; the addition of starch increases the degree of crystallinity of PLA. Therefore, PLA–chitin–starch has values in between the other two composites.

### 3.3. Dynamic Mechanical Analysis

**Figure 2:**
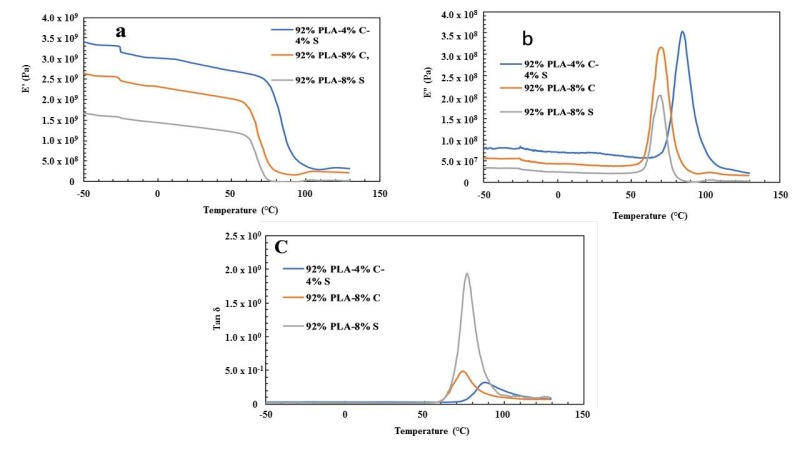
(**a**) Storage modulus (E’); (**b**) loss modulus (E”); (**c**) loss factor tan δ for 92% PLA–4% chitin–4% starch, 92% PLA–8% chitin and 92% PLA–8% starch.

**Figure 3:**
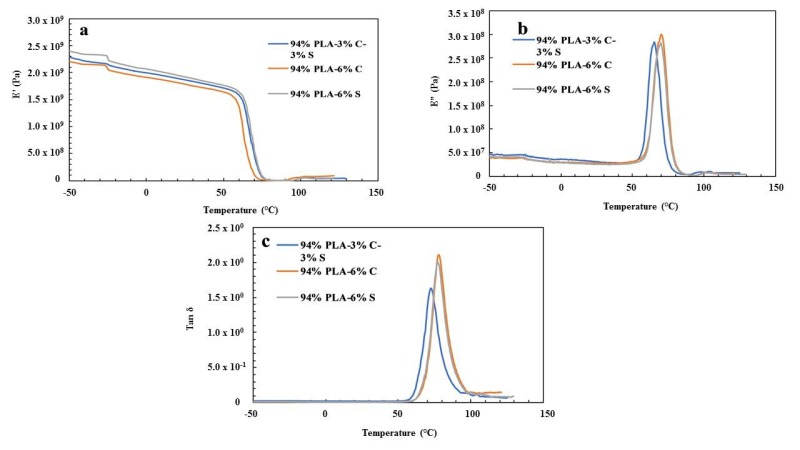
(**a**) Storage modulus (E’); (**b**) loss modulus (E”); (**c**) loss factor tan δ for 94% PLA–3% chitin–3% starch, 94% PLA–6% chitin and 94% PLA–6% starch.

**Figure 4:**
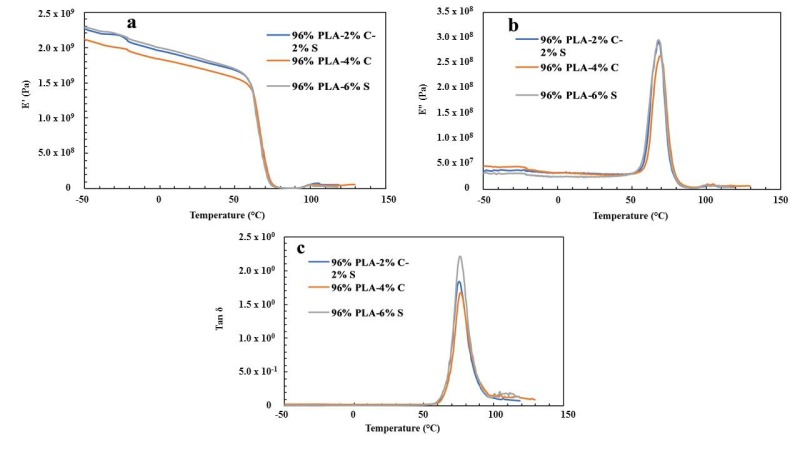
(**a**) Storage modulus (E’); (**b**) loss modulus (E”); (**c**) loss factor tan δ for 96% PLA–2% chitin–2% starch, 96% PLA–4% chitin and 96% PLA–4% starch.

**Figure 5:**
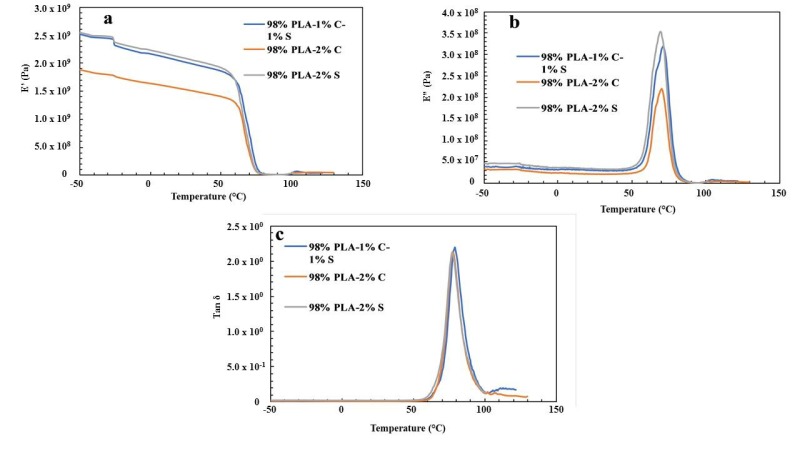
(**a**) Storage modulus (E’); (**b**) loss modulus (E”); (**c**) loss factor tan δ for 98% PLA–1% chitin–1% starch, 98% PLA–2% chitin and 98% PLA–2% starch.

The result of the dynamic mechanical analysis for the twelve samples is shown in Figure 2, Figure 3, Figure 4 and Figure 5. The DMA analysis for 92% PLA–4% chitin–4% starch, 92% PLA–8% chitin and 92% PLA–8% starch is shown in Figure 2a–c. The storage modulus value is seen to be high for 92% PLA–4% chitin–4% starch compared to other samples with an unusual high glass temperature from the loss modulus and loss factor. This could be as a result of uneven distribution of the polymer blend. This is therefore believed to be responsible for the low mechanical strength recorded for the tensile, yield and Young’s modulus as presented in Table 2.

Figure 3a–c shows the storage modulus (E’), loss modulus (E”) and loss factor (tan δ) for 94% PLA–3% chitin–3% starch, 94% PLA–6% chitin and 94% PLA–6% starch, respectively. Generally, the curve shows a typical amorphous characteristic of a polymer composite with a significant drop in the storage modulus at the glass temperature. Also, a single glass temperature for the polymer blends indicates that the three polymers are highly miscible. Since the material blend did not show more than one damping peak, it shows the compatibility of the blend [27]. The value of the storage modulus is directly proportional to the stiffness of the material. The value (Figure 3a) of the storage modulus (E’) for 94% PLA–6% starch is found to be highest at the glassy and rubbery region, which indicates that 94% PLA–6% starch is more brittle than other samples.

The loss modulus indicates heat dissipated per unit deformation, and its peak is referred to as the glass temperature, Tg. The loss modulus and loss factor curve (Figure 3b,c) show a single peak, which indicates the high miscibility of the polymers. The peak of the tan delta curve represents the midpoint of the glass transition temperature Tg [28]. The Tg is one of the significant properties of a polymer composite which determine its application. The value of the glass transition temperature from the loss factor graph is shown in Table 3. A significant increase is noticed in the glass transition temperature for all the samples compared to neat PLA. This increase in the value of Tg is an indication of improvement in the intermolecular bonding within the composite. The trend in storage modulus, loss modulus and loss factor are the same for samples with 96% and 98% PLA in Figure 4 and Figure 5, respectively. The exception is that the storage modulus for 96% PLA–4% starch is more than the other composites at the glassy region, but blends up with the other composites at the transition and rubbery region. This shows that the brittleness is retained at a lower temperature. Also, from the loss modulus and the loss factor curves, the glass transition temperature (Tg) is found not to be affected significantly by the composition variation of the polymers. Table 4 shows the glass transition for the twelve samples.

### 3.4. Thermogravimetry Analysis

**Figure 6:**
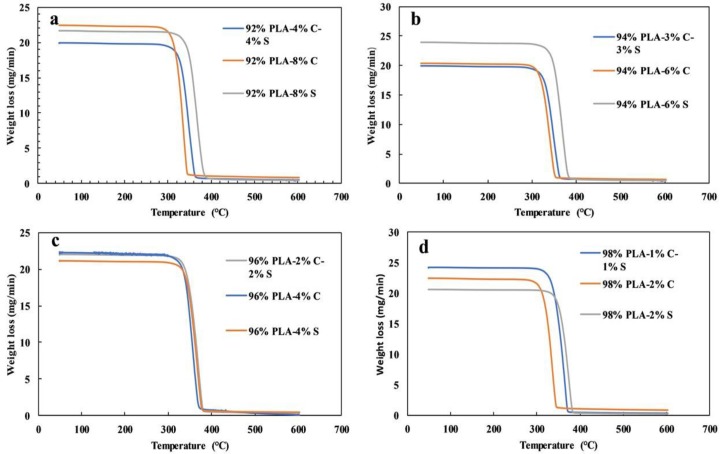
Thermogravimetry analysis for (**a**) 92%, (**b**) 94%, (**c**) 96% and (**d**) 98% PLA sets of samples.

**Figure 7:**
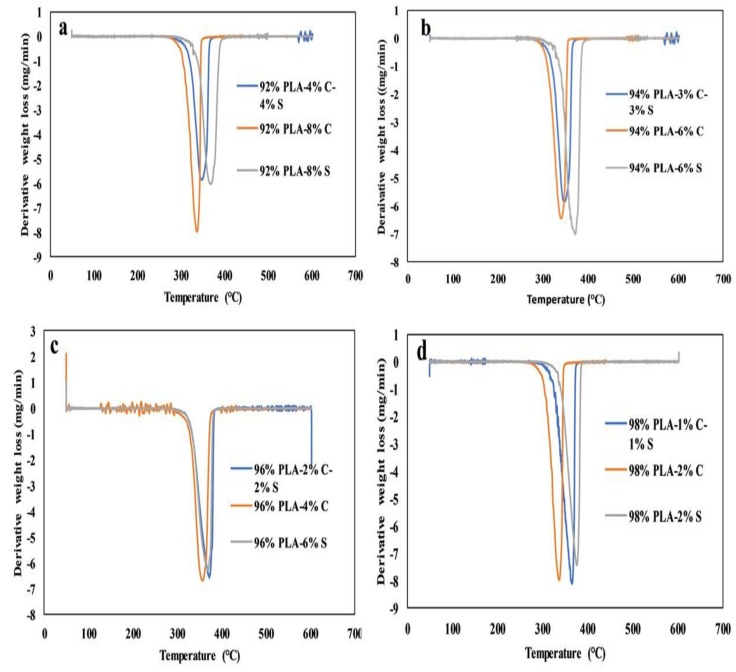
Derivative Thermogravimetry Analysis (DTA) for (**a**) 92%, (**b**) 94%, (**c**) 96% and (**d**) 98% PLA sets of samples.

The Thermogravimetry Analysis (TGA) and Derivative Thermogravimetry Analysis (DTA) results for the twelve samples are shown in Figure 6a–d and Figure 7a–d. The thermal stability of pure PLA–chitin–starch, PLA–chitin, and PLA–starch are similar for the twelve samples. Generally, the TGA curves have a single thermal degradation from the weight loss with a definite peak temperature on the DTA curves. This implies that the polymers are compatible with each other with no segregation. Like the DMA curves, the PLA–starch curves have the highest weight loss and it is more significant with 94% PLA curves, with obvious reduction as the percentage of PLA increases to 98%.

The change in composition can be seen to affect the melting peaks in the DTA curves and it is more obvious in the 94% PLA and 98% PLA set of samples, but lower for the 96% samples. As evident in Figure 6a, 92% PLA–8% chitin started to decompose around 336 °C, whereas 92% PLA–4% chitin/4% starch and 92% PLA–8% starch started around 343 °C and 369 °C, respectively. Also, a similar trend can be seen for 94%, 96% and 98% PLA samples as 94% PLA–6% chitin, 94% PLA–3% chitin–3% starch, and 94% PLA–6% starch started to decompose at 292 °C, 313 °C, and 322 °C, respectively; 96% PLA–4% chitin, 96% PLA–2% chitin–2% starch, and 96% PLA–4% starch at 289 °C, 287 °C, and 315 °C, respectively; and 98% PLA–2% chitin, 98% PLA–1% chitin–1% starch, and 98% PLA–2% starch at 292 °C, 312 °C, and 326 °C, respectively. The curves do not show much difference as they are close to each other with similar path behavior with respect to the significant drop in weight, which is mainly due to material degradation. As the process approached transition, the curves cannot be distinctively differentiated, but distinctive peaks could be seen on the DTA curves. This behavior is also seen for the degradation products, which is probably due to the formation of char. Generally, the thermal stability of the PLA blends with chitin and starch seems to improve with the addition of starch content and reduce with chitin content. This improvement in the thermal stability is evident in the DTA melting peaks. It is however worthy of note that the thermal stability of the blend decreased with an increase in the percentage of PLA. However, the nature of the TGA and DTA curves shows that variation in composition has no significant influence on the degradation behavior of the composite (compared with neat PLA). This observation is similar to what has been previously reported [25,29].

The miscibility can be explained using the nature of the polymer blends. PLA is hydrophobic, whereas starch is hydrophilic, which makes them thermodynamically immiscible [30]. However, the miscibility of the blend can be explained from the works of [30,31]. It was stated that the improvement in the adhesion properties of PLA–starch can be achieved using a material with strong compatibility with PLA and starch [31]. Chitin fits suitably into this category based on its excellent adhesion with PLA [25,32] and starch [33]. Therefore, the chitin in this blend serves as an interfacial compatibilizer between PLA and starch, bridging inter-facial transitions [31,34].

### 3.5. Scanning Electron Microscopy

**Figure 8:**
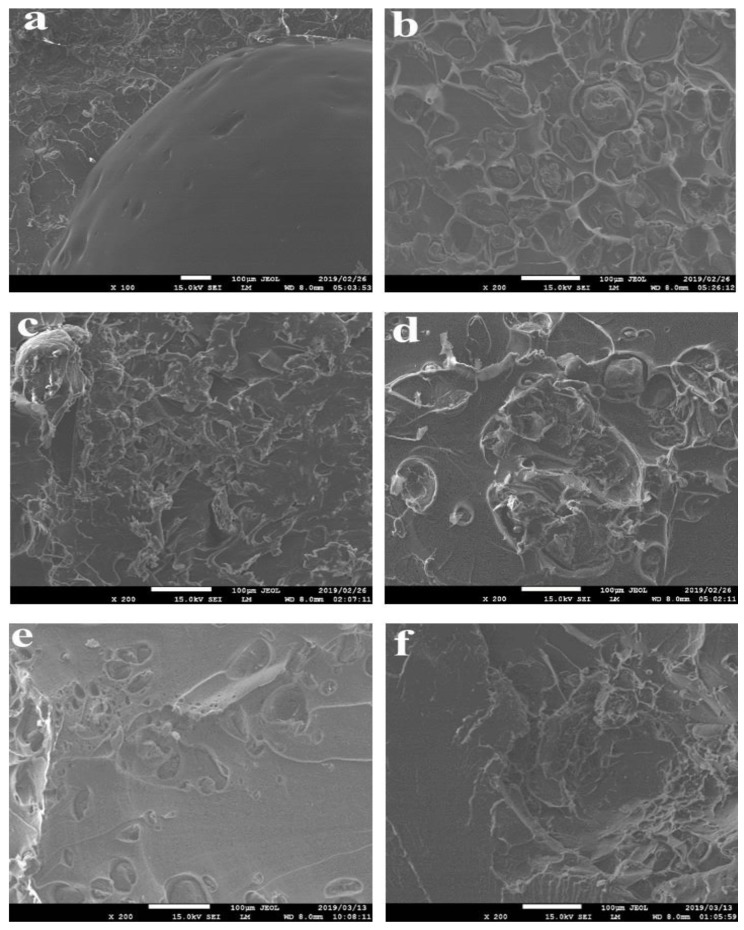
Scanning electron microscopy (SEM) fracture images. (**a**) 92% PLA–4% chitin–4% starch; (**b**) 92% PLA–8% chitin; (**c**) 92% PLA–8% starch; (**d**) 94% PLA–3% chitin–3% starch; (**e**) 94% PLA–6% chitin; (**f**) 94% PLA–6% starch.

**Figure 9:**
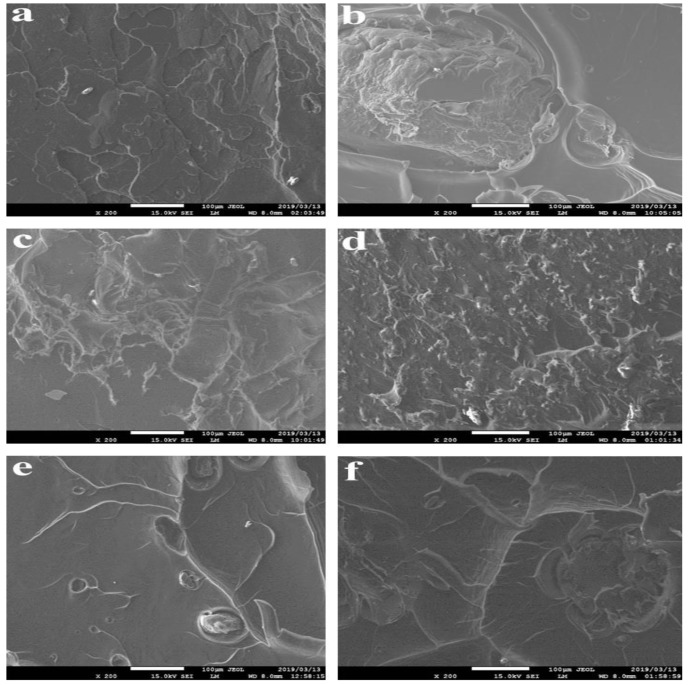
SEM fracture images. (**a**) 96% PLA–2% chitin–2% starch; (**b**) 96% PLA–4% chitin; (**c**) 96% PLA–4% starch; (**d**) 98% PLA–1% chitin–1% starch; (**e**) 98% PLA–2% chitin; (**f**) 98% PLA–2% starch.

**Figure 10:**
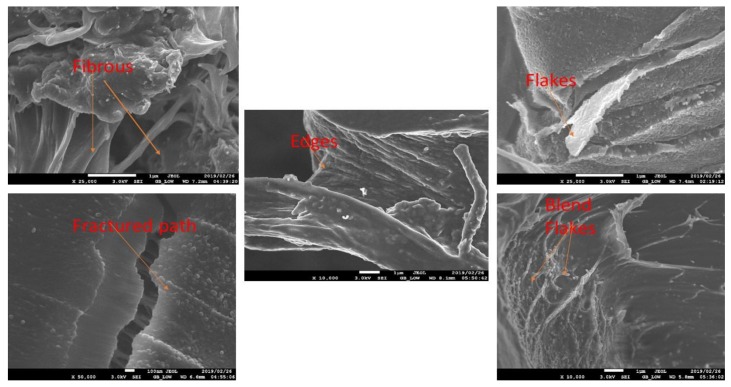
SEM fracture images of high magnification PLA–chitin–starch.

The fractured surface images of the samples are presented in Figure 8, Figure 9 and Figure 10. The properties of each sample are dependent on the extent of dispersion blends within the composite. In general, the SEM images show that a good dispersion is achieved using the methodology of double-step mixing using a rheomixer and an extruder. There is a bright appearance of compatibility or adhesion of the three-polymer blend in the PLA–chitin–starch blend as well as in the PLA–chitin and PLA–starch samples. The tensile fracture surface morphology for the PLA–chitin–starch (Figure 8a,d and Figure 9a,d) and PLA–starch (Figure 8c,f and Figure 9c,f) samples reveals a network of a well-dispersed blending of chitin and starch, respectively, with few voids on the fractured surface. The bridging effect of the networks prevents cracking and enables effective stress transfer [29]. PLA–chitin (Figure 8b,e and Figure 9b,e) has a smoother surface with fewer voids when compared to the that of PLA–chitin–starch and PLA–starch. This can be explained by the thermal degradation curve which shows the improvement in the degradation property of PLA, and this is also documented in the reports of [16] and [25]. This is more significant in the 98% PLA–2% chitin sample with no noticeable void. According to previous research, chitin is mainly used to improve the degradation properties of PLA, and this suggests a better chemical interaction between PLA and chitin blends [27]. 

The SEM images shows an uneven distribution in the 92% PLA–4% chitin–4% starch sample (Figure 8a), which is probably responsible for the low mechanical strength. Generally, the composite samples for 94% (Figure 8d–f), 96% (Figure 9a–c) and 98% (Figure 9d–f) PLA is accompanied with a network of dispersion which peaks at 98% with smooth co-continuous morphology with flakes and less defined edges [27]. The dispersion network may be responsible for the better mechanical performance, especially considering the enhanced intermolecular bonding as evident in the network. Also, the PLA–chitin blend shows a lesser dispersed network dominated by PLA. The morphology shows high mixing in the blend such that presence of chitin is not noticeable. This suggests adhesion and good interaction. PLA–starch, on the other hand, has significant edges and wedges with good segmental dispersion. Higher magnification of the PLA–chitin–starch blend is shown in Figure 10.

## 4. Conclusions

The comparative analysis of the effect of PLA blending with chitin and starch shows that PLA–chitin–starch composites are miscible with each other without segregation of the particles. The composite, molded using two-step mixing and a Carver Press, results in a more uniform composite. The mechanical properties of the polymer composites increased significantly compared to pure PLA for all samples. The mechanical strength result of PLA–chitin–starch revealed a significant improvement compared with binary blends such as PLA–chitin and PLA–starch. However, the dynamic mechanical properties of the composites showed notable improvement with an increase in chitin content and decrease in starch content. Also, the DMA result shows the high amorphous flow of the polymer blend with excellent mechanical strength over a range of temperatures. The TGA/DTA result shows one single degradation curve with a single glass temperature, which conforms with the DMA result and indicates miscibility and compatibility of the three polymers. The SEM images show excellent blending properties with a dispersed network of starch and chitin. Overall, the combined effect of PLA–chitin–starch gave a better result than PLA–chitin or PLA–starch. 

## Figures and Tables

**Table 1:** Particle size distribution. PLA = poly(lactic acid).

Particle Size	<1 µm	1–45 µm	46–75 µm	76–150 µm	151–500 µm	501–700 µm	>700 µm
PLA %	0.02	0.03	11.24	18.50	3.45	6.81	59.95
Chitin %	71.2	0.28	25.6	0.9	1.56	0.46	0
Starch%	11.01	34.8	49.64	0.31	4.24	0	0

**Table 2:** Composition variation.

Composite	Proportion (wt.%)			
**PLA–chitin**	92:8	94:6	96:4	98:2
**PLA–starch**	92:8	94:6	96:4	98:2
**PLA–chitin–starch**	92:4:4	94:3:3	96:2:2	98:1:1

**Table 3:** Tensile properties of PLA–chitin (C)–starch (S) composites.

Samples	Tensile Modulus	Yield Strength	Elongation (%)	Tensile Strength
	(MPa)	(MPa)		(MPa)
92% PLA–4%C–4% S	2138.1 ± 107.3	75.3 ± 2.9	4.3 ± 0.3	75.3 ± 2.92
92% PLA–8% C	2898.6 ± 104.9	82.8 ± 2.6	5.5 ± 0.7	82.9 ± 2.26
92% PLA–8% S	2412.2 ± 117.9	82.8 ± 2.1	4.1 ± 0.3	82.8 ± 2.12
94% PLA–3% C–3% S	2458.1 ± 108	84.4 ± 1.7	5.0 ± 0.9	84.4 ± 1.7
94% PLA–6% C	2310.7 ± 101.6	83.0 ± 1.6	7.1 ± 1.4	83.0 ± 1.6
94% PLA–6% S	2417.3 ± 101.6	70.6 ± 1.9	3.9 ± 0.3	70.6 ± 1.9
96% PLA–2% C–2% S	2613.3 ± 103.3	83.0 ± 1.3	5.3 ± 1.8	83.0 ± 1.3
96% PLA–4% C	2448.9 ± 102.1	80.8 ± 1.9	6.3 ± 2.2	80.8 ± 1.9
96% PLA–4% S	2586.4 ± 106	79.7 ± 1.4	4.2 ± 0.4	79.7 ± 1.4
98% PLA–1% C–1% S	2536.3 ± 108.3	82.6 ± 2.9	4.8 ± 0.4	82.6 ± 2.9
98% PLA–2% C	2524.0 ± 105.8	77.4 ± 2.1	5.5 ± 1.2	77.4 ± 2.0
98% PLA–2% S	2058.4 ± 104.3	72.1 ± 1.5	4.2 ± 0.3	72.1 ± 1.5
100% PLA	3500.0 ± 100.1	53.5 ± 1.1	6.0 ± 0.2	60.0 ± 1.12

**Table 4:** Glass transition temperature for the samples.

Serial Number	Samples	Glass Transition Temperature
		(°C)
1	92% PLA–4% C–4% S	73.0
2	92% PLA–8% C	74.0
3	92% PLA–8% S	91.6
4	94% PLA–3% C–3% S	75.5
5	94% PLA–6% C	76.4
6	94% PLA–6% S	76.7
7	96% PLA–2% C–2% S	67.0
8	96% PLA–4% C	69.8
9	96% PLA–4% S	76.7
10	98% PLA–1% C–1% S	77.2
11	98% PLA–2% C	76.9
12	98% PLA–2% S	76.6
13	100% PLA	60.0
